# Donors for nerve transplantation in craniofacial soft tissue injuries

**DOI:** 10.3389/fbioe.2022.978980

**Published:** 2022-09-07

**Authors:** Sishuai Sun, Di Lu, Hanlin Zhong, Chao Li, Ning Yang, Bin Huang, Shilei Ni, Xingang Li

**Affiliations:** ^1^ Department of Neurosurgery, Qilu Hospital, Cheeloo College of Medicine and Institute of Brain and Brain-Inspired Science, Shandong University, Jinan, China; ^2^ Jinan Microecological Biomedicine Shandong Laboratory and Shandong Key Laboratory of Brain Function Remodeling, Jinan, China

**Keywords:** soft tissue injuries, nerve transplantation, Biomaterials, tissue engineering, Regenerative medicine, nerve conduit, stem cell, 3D printing

## Abstract

Neural tissue is an important soft tissue; for instance, craniofacial nerves govern several aspects of human behavior, including the expression of speech, emotion transmission, sensation, and motor function. Therefore, nerve repair to promote functional recovery after craniofacial soft tissue injuries is indispensable. However, the repair and regeneration of craniofacial nerves are challenging due to their intricate anatomical and physiological characteristics. Currently, nerve transplantation is an irreplaceable treatment for segmental nerve defects. With the development of emerging technologies, transplantation donors have become more diverse. The present article reviews the traditional and emerging alternative materials aimed at advancing cutting-edge research on craniofacial nerve repair and facilitating the transition from the laboratory to the clinic. It also provides a reference for donor selection for nerve repair after clinical craniofacial soft tissue injuries. We found that autografts are still widely accepted as the first options for segmental nerve defects. However, allogeneic composite functional units have a strong advantage for nerve transplantation for nerve defects accompanied by several tissue damages or loss. As an alternative to autografts, decellularized tissue has attracted increasing attention because of its low immunogenicity. Nerve conduits have been developed from traditional autologous tissue to composite conduits based on various synthetic materials, with developments in tissue engineering technology. Nerve conduits have great potential to replace traditional donors because their structures are more consistent with the physiological microenvironment and show self-regulation performance with improvements in 3D technology. New materials, such as hydrogels and nanomaterials, have attracted increasing attention in the biomedical field. Their biocompatibility and stimuli-responsiveness have been gradually explored by researchers in the regeneration and regulation of neural networks.

## Introduction

Although the surface area of the head and neck accounts for approximately 12 percent of the entire body, they are widely populated with craniofacial nerves, which perform various functions. As part of peripheral nerves, craniofacial nerves are responsible for extremely complex sensory and motor functions, including olfaction, vision, hearing, taste, touch, chewing, eye movement, and balance. It is important to pay attention to nerve repair during craniofacial injuries. The causes of craniofacial nerve injury are complex and diverse. Trauma, infection, inflammation, tumors, and iatrogenic factors can cause reversible or irreversible nerve injuries (Jandali and Revenaugh, 2019; Pan et al., 2020). Therefore, the complicated anatomical structure and physiological functions of the craniofacial nerves make their regeneration a challenge.

In 1943, Herbert Seddon proposed a classification method based on the severity and prognosis of peripheral nerve injuries, including neurapraxia, axonotmesis, and neurotmesis (Kaya and Sarikcioglu, 2015). Based on Seddon’s classification, Sunderland proposed five categories according to the extent of axonal injury (Sunderland, 1951) (Table 1). The degree and type of nerve injury should be considered in surgical repair. The location, length, shape, and cross-sectional area of the donor and the damaged nerves are important considerations in donor selection. Patient preferences should be assessed, as nerve harvesting may result in loss of function at the donor site (Altafulla et al., 2019). However, autologous nerve transplantation is still the preferred treatment for segmental nerve defects that are difficult for primary repair. However, imperfect results have suppressed the advantages of autologous transplantation as the first-line option, which include the formation of neuroma, loss of sensation at the donor site, infection, and pain caused by the nerve stump. This has encouraged researchers to explore additional treatment options for nerve transplantation (Szynkaruk et al., 2013; Carvalho et al., 2020). Allotransplantation allows a wide range of donor sources for nerve transplantation. Among these, the complex functional unit containing nerve tissue facilitates nerve function recovery in multiple tissue injuries. Meanwhile, there is the risk of long-term immunosuppression after transplantation and infection, especially under circumstances where the structures of cranial and facial tissues are complex and the bacterial flora is diverse (Husain et al., 2003; Singh et al., 2005; Cuellar-Rodriguez et al., 2009). In recent years, the advantages of nerve conduits combined with tissue engineering have been realized in the field of nerve transplantation (Zor et al., 2010; Szynkaruk et al., 2013; Roche et al., 2015a; Long et al., 2021). Researchers are incorporating absorbable and non-absorbable materials, such as silica gel, polyhydroxy acid, poly (lactate-glycolic acid) graphene foam, and polycaprolactone, in these new conduits (Han et al., 2019; Bahremandi Tolou et al., 2021). Improving the physiological performance of emerging grafts by combining cells, such as Schwann cells (SC), or growth factors has become an important research trend (Huard et al., 1998; Evans, 2001; Murrell et al., 2005; Matsumine et al., 2016; Watanabe et al., 2017). These new explorations have stimulated the interest of researchers in using novel materials, such as hydrogels and nanomaterials, for nerve regeneration (Oprych et al., 2016; Celikkin et al., 2017; Fan et al., 2017). Their superior biocompatibility allows simulation of the extracellular matrix (ECM) and microenvironment *in vivo* to promote adhesion and survival of neural cells. Sensitive responses to external stimuli make it possible to use external factors (light, electricity, magnetic field, temperature, etc.) to achieve the directional control of implant materials (Riggio et al., 2014; Marcus et al., 2016; Abatangelo et al., 2020).

**TABLE 1:** Seddon’s and Sunderland’s classification of peripheral nerve injuries. Peripheral nerve injuries are divided into three categories in Seddon’s classification, including neurapraxia, axonotmesis and neurotmesis. Based on Seddon’s classification, Sunderland classified five different levels in detail according to the extent of a xonal injury. Neurapraxia in Seddon’s classification is equivalent to the first degree injury in Sunderland’s classification. Neurotmesis is equivalent to the fifth degree nerve injury and axonotmesis is equivalent to the second-to-fourth-degree nerve injury.

Seddon’s Classification	Sunderland’s classification	Axon	Myelin	Endoneurium	Perineurium	Epineurium
Neurapraxia	1st-degree	**√** ^a^	×	√	√	√
	2nd-degree	×^b^	×	√	√	√
Axonotmesis	3rd-degree	×	×	×	√	√
	4th-degree	×	×	×	×	√
Neurotmesis	5th-degree	×	×	×	×	×

a Intact.

b Damaged or severed.

**FIGURE 1:**
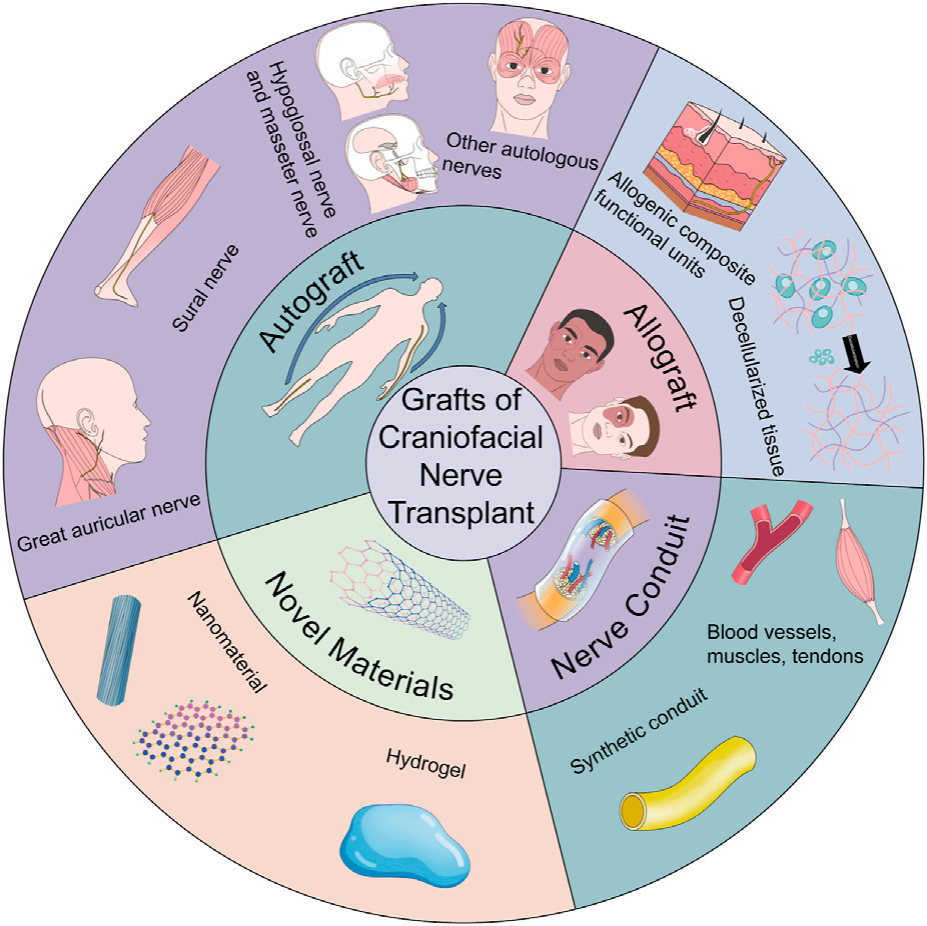
The brief description on donors for nerve transplantation in craniofacial soft tissue injuries.

To drive cutting-edge research on craniofacial nerve repair and its progression from the laboratory to the clinic, we reviewed the selection of donors for repair and regeneration of craniofacial nerve injury, especially segmental defects. The main focus is on nerves that are easily damaged and have obvious effects on human function, such as the facial nerve, trigeminal nerve and its branches (mandibular nerve, inferior alveolar nerve), branches of the vagus nerve (recurrent laryngeal nerve), optic nerve, and oculomotor nerve. The present article has summarized the following areas: autografts, allografts, xenografts, and nerve conduits. Allografts have been subdivided into allogeneic composite functional units and decellularized tissues, as required. Finally, the two most widely used novel materials (hydrogels and nanomaterials) in the biomedical field are described in detail to promote the application of novel materials for craniofacial nerve repair (Figure 1).

## Autografts

Given the underlying principles, end-to-end repair is not feasible for peripheral nerve regeneration in some cases, such as excessive nerve tension, delayed repair, or obvious segmental nerve defects. Autogenous nerve transplantation is the first-line treatment for this condition. In addition to better biocompatibility, autografts can provide cells (e.g., SCs) and cellular components that can promote nerve growth. They have not been associated with rejection and have a lower infection rate than allografts (Moore et al., 2015). However, autografts have some unavoidable disadvantages, including trauma and sensory defects as a result of harvesting the donor nerve and pain caused by the nerve stump. Therefore, the selection of autografts should be based on the principles of easy acquisition, less trauma, and a match with the size at the recipient site (Trehan et al., 2016). Common clinical donors for craniofacial nerve autotransplantation include the great auricular nerve (GAN), sural nerve, hypoglossal and masseter nerves, antebrachial cutaneous nerve, and motor nerve to the vastus lateralis.

### Great auricular nerve

The GAN is a branch of the cervical plexus that arises from the second and third cervical nerves and passes through the posterior margin of the sternocleidomastoid muscle (SCM). It proceeds forward and upward and divides into two branches that are dominant in the areas of the mandible, auricula, and earlobe (Humphrey and Kriet, 2008; Altafulla et al., 2019). GAN has more than one marker. Traditionally, the McKinney spot, which is 6.5 cm below the external auditory canal and 0.5 cm behind the external jugular vein, has been the anatomical marker for distinguishing the GAN during surgical localization (McKinney and Katrana, 1980). The greater auricular point of the GAN at the posterior edge of the SCM has also been described as an important landmark (Raikos et al., 2017). Another marker, Erb’s point, is situated 2–3 cm above the clavicle and at the same level as the carotid nodule (Tubbs et al., 2007a). Researchers have delineated another marker for locating the mastoid branch of the GAN, which is 9 cm outside the external occipital eminence and 1 cm above the mastoid cusp (Tubbs et al., 2007b). Unlike traditional landmarks, this point uses bony structures that are easy to localize rather than deep blood vessels or muscles.

Transplantation of the craniofacial nerve and its branches with the GAN as a donor can be used for several types of nerve repair, including facial nerve repair, jaw reconstruction and restoration of perioral sensation, recurrent laryngeal nerve reconstruction after thyroidectomy to preserve vocal function, promoting corneal nerve regeneration, and restoration of sensation as a surgical method for neurotrophic keratosis (LaBanc et al., 1987; Yoshimura et al., 2013; Sun et al., 2015; Benkhatar et al., 2018). For facial nerve reconstruction, the GAN is used to reconstruct nerve damage caused by resection of craniofacial masses, such as facial nerve schwannomas, endolymphatic sac tumor, facial nerve hemangioma, skull base tumor, and nerve defects or function loss caused by trauma and inflammatory diseases (Stephanian et al., 1992; Katoh et al., 1998; Chi et al., 2006; De Ceulaer et al., 2012; Hou et al., 2012; Marchioni et al., 2014; Sai et al., 2019). Selecting the GAN as the donor for facial nerve repair has advantages, such as proximity to the surgical region, which helps avoid a second site of surgery, and easy localization because it is a clear marker. The GAN is also utilized as a nerve graft to reconstruct the inferior alveolar nerve for mandibular defects resulting from trauma or mandibular resection caused by a tumor. Yoshimura et al. performed segmental mandibular resection of a large ossifying fibroma and immediate reconstruction with the iliac bone and GAN. Postoperative neurosensory examination revealed sensitivity recovery of the dental pulp and mental region (Yoshimura et al., 2013). GAN transplantation for repairing craniofacial nerve defects has shown relatively stable effects in long-term clinical applications; the most commonly used nerve donor is the GAN without blood vessels.

However, vascularized nerve grafts may promote nerve regeneration better than non-vascularized nerve grafts when they are longer and nerve recovery conditions are poor (e.g., in areas with poor vascularization). Koshima et al. used a vascularized GAN graft for the primary repair of facial nerve defects. The nerve regeneration results were satisfactory, and there were fewer complications at the donor site. The main reason for the better effects of this transplantation is that blood supply can be restored immediately after surgery using a vascularized nerve graft (Koshima et al., 2004). Therefore, vascularized grafts have more significant early effects on nerve repairs. Corneal nerve fibers arise predominantly from the ophthalmic branch of the trigeminal nerve. Neurotrophic keratitis primarily results from herpes virus invasion, corneal surgery, diabetes, and orbital or intracranial surgery-related trigeminal nerve damage. Corneal neuralization is increasingly being used to restore corneal innervation after trigeminal nerve injury (Benkhatar et al., 2018). End-to-end anastomosis of the GAN and contralateral supratrochlear nerve (Benkhatar et al., 2018) or implantation of sensory fibers from the ipsilateral GAN in the precorneal stroma (Jowett and Pineda Ii, 2019) is effective in restoring corneal perception in adults.

### Sural nerve

The sural nerve is a sensory nerve consisting of branches of the tibial nerve and common peroneal nerve. It passes through the deep fascia and descends laterally to the Achilles tendon before advancing to the lateral malleolus and the distal side of the calcaneus (Im et al., 2022). In craniofacial nerve transplantation, sural nerves are often used to restore sensory disturbances and malocclusions in the mandibular region as a result of inferior alveolar nerve (IAN) defects caused by tumor resection or trauma (LaBanc et al., 1987; DeLeonibus et al., 2017) and facial paralysis caused by facial nerve injury (Biglioli et al., 2018; Jeong et al., 2018; Baccarani et al., 2019; Rashid et al., 2019; Yoshioka, 2020; Chang et al., 2021; Sakuma et al., 2021). Traditionally, end-to-end suturing of the sural nerve with the nerve stump via interposition transplantation has been a common method for repairing nerve defects (Rashid et al., 2019; Matos Cruz and De Jesus, 2022). Rashid et al. used sural nerves to repair facial nerve defects caused by parotid gland tumor resection. The results demonstrate that sural nerve interposition transplantation is easy to perform and has a good effect on segmental facial nerve defects (Rashid et al., 2019). However, nerve sharing or cross nerve transplantation shows greater advantages when the proximal end of the recipient nerve is not available or the defect site is too long. LaBanc et al. reported a case of the successful restoration of the sensation of the lower lip by transplanting the autogenous sural nerve to the region between the ipsilateral GAN and distal IAN using the principle of nerve sharing (LaBanc et al., 1987). In 2009, researchers presented a new method of repairing the paralyzed eyelid, which involves transferring the facial nerve on the undamaged side to supply the upper and lower eyelids with two sural nerve grafts. Biglioli modified this technique by repairing the upper eyelid using cross grafting of the facial and sural nerves, with the lower eyelid suspended upward by the fascia lata. Eyelid closure and the blink reflex were effectively observed in 14 patients with eyelid paralysis who underwent this surgery (Biglioli et al., 2018). Therefore, nerve sharing and cross nerve transplantation have shown significant advantages in patients with long nerve defects. The muscle-nerve-muscle neurotisation technique also embodies the principle of nerve sharing. For example, the sural nerve was attached to the bilateral frontalis muscle to facilitate innervation from the healthy to the affected frontalis muscle. Postoperative nerve conduction and muscle response were normal, and spontaneous and simultaneous contraction of the bilateral frontalis muscles was successfully observed 4 months after surgery (Chang et al., 2021). Similar to the GAN, the sural nerve plays a significant role during corneal neuralization in neurotrophic keratopathy and can successfully promote the restoration of corneal sensation (Weis et al., 2018; Elalfy et al., 2021; Charlson et al., 2022). However, previous studies have found that there is no obvious distinction between the repair ability of the sural nerve and GAN for corneal neuralization; however, it can provide more options for adult patients with neurotrophic keratopathy. In this situation, the patient’s choice and physical condition may be decisive factors.

### Hypoglossal nerve and masseter nerve

The hypoglossal and masseter nerves have similar functions in nerve transplantation but are not as widely used as sural nerves and the GAN, which are mainly used as donors for facial nerve anastomosis in facial resuscitation (Bayrak et al., 2017; Yetiser, 2018; Yoshioka, 2018). Anastomosis of the hypoglossal and facial nerves is one of the earliest methods used for facial resuscitation. Complete disconnection of the hypoglossal nerve and anastomosis with the main trunk of the facial nerve are the main steps during the surgery. These can provide sufficient facial tension and motor stimulation but may result in lateral tongue atrophy and difficulties with speech, chewing, and swallowing (Jandali and Revenaugh, 2019). However, anastomosis of the masseter and facial nerves can reduce the postoperative complications of dysarthria and dysphagia (Bayrak et al., 2017). The masseter nerve has several advantages, such as a stronger motor impulse, fewer complications of the donor defect, and faster recovery of innervation (Sakthivel et al., 2020). Therefore, the masseter nerve presents greater advantages than the hypoglossal nerve as a donor for facial nerve anastomosis. However, a single transfer of the hypoglossal or masseteric nerve can cause significant postoperative mass movement because both are single motor sources reinnervating the entire muscular system (Yoshioka, 2018). The joint transfers between the hypoglossal and masseteric nerves may solve this problem and promote nerve repair and motor coordination. Kuta et al. used the masseter nerve to selectively innervate the midface and restore the vitality of the remaining region of the face in combination with hypoglossal nerve transplantation. Apparent improvements in facial movement and resting facial tone were observed without mass movement after surgery (Kuta and Taylor, 2022). Yoshioka N et al. performed joint nerve transfers and cross grafting of the facial nerves. The masseter and hypoglossal nerves were transferred to the zygomatic and cervicofacial branches of the facial nerves, respectively. Patients obtained symmetrical resting lips and could raise their mouth corners voluntarily without impaired chewing function, tongue atrophy, or significant mass movement (Yoshioka, 2018). Therefore, double nerve transfer involving the hypoglossal and masseter nerves has more advantages in restoring facial tension, symmetry, and coordinated movement.

### Other autologous nerves

In addition to the commonly used donor nerves mentioned above, the antebrachial cutaneous nerve and motor nerve to the vastus lateralis also have the potential to be used in the repair of the craniofacial nerve, especially for facial nerve repair (Revenaugh et al., 2012; Jandali and Revenaugh, 2019). Rodriguez-Lorenzo et al. observed complete restoration of nerve function after using the platysma motor nerve to repair the segmental defect of the marginal mandibular nerve caused by resection of soft tissue sarcoma of the jaw (Rodriguez-Lorenzo et al., 2016). This approach has been proven to be more effective in recovering laryngeal local function than performing laryngeal reinnervation utilizing the ansa cervicalis, but it has not shown significant advantages compared with the traditional vocal fold medialization laryngoplasty. Comparative studies are underway (Paniello et al., 2011; Wang et al., 2011). Li et al. reconstructed the parotid gland defects by innovatively constructing the vascularized latissimus dorsi nerve flap. The facial symmetry and smiling improved postoperatively, and it was confirmed that vascularized nerve donors can induce better nerve recovery than single nerve donors (Li S. S. et al., 2021). The supratrochlear or supraorbital nerve has also been reported for corneal neutralization. However, using the GAN can minimize further loss of facial sensation in persons who have suffered sensory deficits relative to the transfer of the supratrochlear or supraorbital nerves. However, this carries the risk of a sub-optimal blink reflex recovery (Jowett and Pineda Ii, 2019). In general, more clinical cases and research are needed to confirm the advantages and disadvantages of these rare or modified grafts as alternatives to traditional transplant donors.

## Allografts

### Allogeneic composite functional units

**FIGURE 2:**
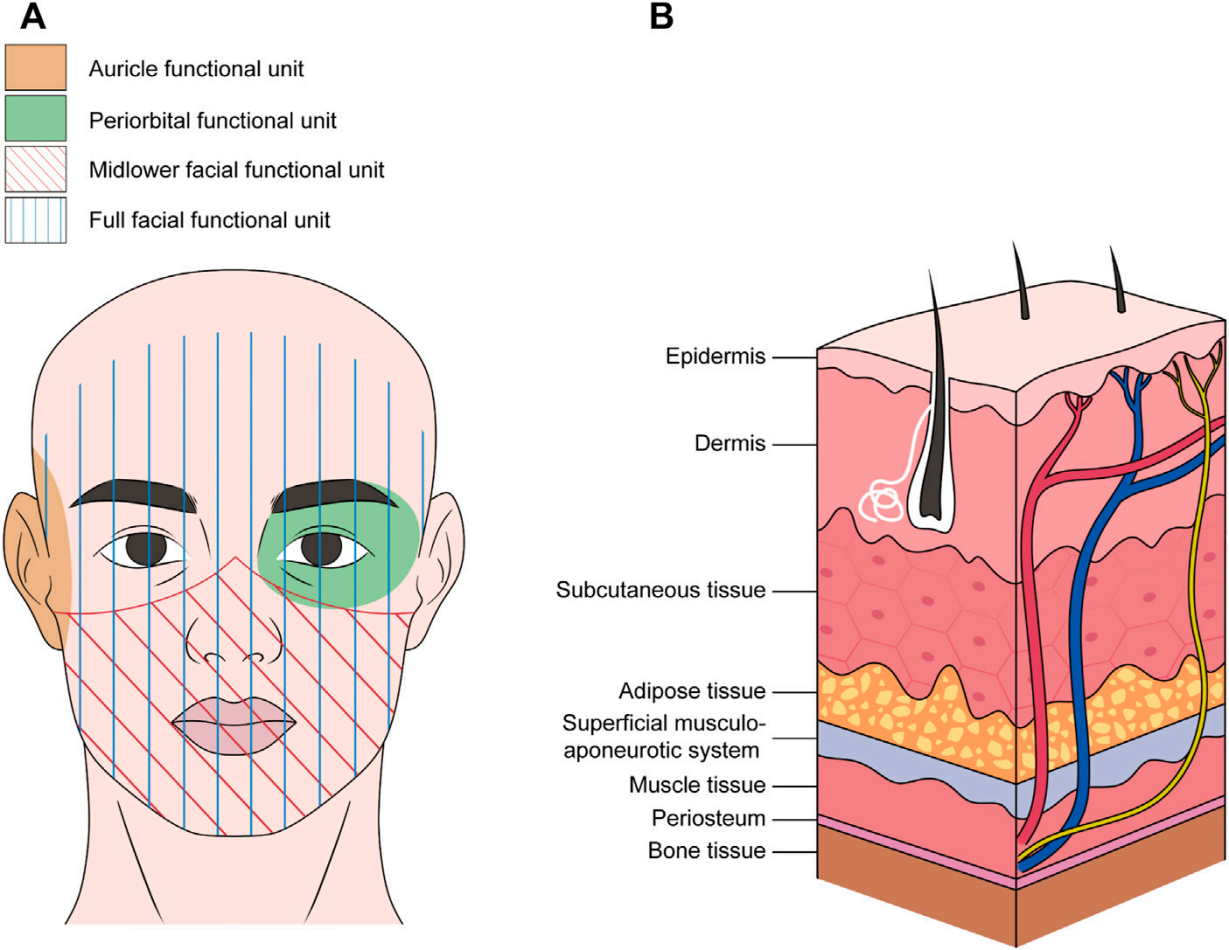
Common craniofacial allogeneic composite functional units **(A)** and tissue composition **(B)**.

Craniofacial nerve composite allograft transplantations are used to repair severe facial injuries such as severe trauma, deep burns, and multiple tissue defects after tumor resection. The ideal reconstructive surgery allows the repair of damaged tissue and restoration of motor and sensory functions to promote optimal functional recovery of the recipient site. However, autologous transplantation does not guarantee ideal functional and aesthetic recovery of complex craniofacial defects (Devauchelle et al., 2006). Therefore, optimizing nerve regeneration by utilizing allogeneic composite functional units is beneficial for overall functional recovery (Zor et al., 2010; Roche et al., 2015a). In allogeneic composite functional units, nerve components are part of the functional unit rather than independent grafts. The functional unit includes the skin, muscle, bone, fat, and lymph nodes, in addition to nerve tissue (Broyles et al., 2014). The midface, lower face, auricle, and periorbital and perinasal regions are common functional units (Figure 2). The main nerves involved are the trigeminal and facial nerves and their branches, such as the maxillary, mandibular, buccal, and zygomatic branches of the facial nerve (De Letter et al., 2017; Lassus et al., 2018; Kauke et al., 2021). Devauchelle et al. were the first to perform the human face allograft. In addition to soft tissue reconstruction, the bilateral infraorbital and mental-sensitive nerves were anastomosed. The mandibular branch of the facial nerve was used to innervate the mimic muscle. Postoperative sensory function and verbal ability improved rapidly, and the muscle contractile function gradually recovered. These results not only verify the feasibility of allogeneic complex tissue transplantation for repairing severe facial injury but also provide important technical guidance for the extensive development of human craniofacial allografts (Devauchelle et al., 2006). At present, the consistency of the donor and injury sites is the main consideration in the selection of transplant units for composite transplantation. For example, a patient suffered a severe facial defect, resulting in a large soft tissue defect on one side of the face with severe damage to the upper jaw, both eyes, bottom of the mouth, and left side of the jaw, and all the teeth. After the clinical evaluation, the lower parts of the face were used as functional units for transplantation. The main nerves of the unit contain the facial nerves and branches. In addition, the functional unit includes the maxilla, mandible, blood vessels, and skin of the middle and lower two-thirds of the face (Roche et al., 2015b). Eyelid defects for various reasons often cause chronic eyelid discomfort, corneal ulcers, and visual impairment. Complex ocular and periorbital defects may occur after traumatic avulsion, burns, or cancer resection. It is difficult to achieve better functional and aesthetic restorations using traditional reconstruction methods (Vasilic et al., 2010; Siemionow et al., 2018). In this case, a periorbital functional unit allograft may be a better option. The researchers used fresh cadavers to develop a new model of composite eyeball-periorbital transplants. The optic nerve, oculomotor nerve, and trochlear nerve were included to promote the recovery of visual acuity and eye movement. The edge of the composite flap included the lower and lateral margins of the orbit, nasal dorsum, and the upper edge of the eyebrow. This study confirmed the feasibility of composite eyeball-periorbital transplantation and provided a new option for the reconstruction of complex periorbital malformations, as well as a new vascularized composite allograft model for whole-eye transplantation (Siemionow et al., 2018). In brief, as the donor of a craniofacial nerve allograft is mostly a composite functional unit, the main requirement for selection is consistency with the injury site. Currently, there are more preclinical studies on the functional units of craniofacial nerve transplantation, particularly facial transplantation. However, these studies have mainly focused on the feasibility of functional units centered on single organs, such as periorbital and auricular functional units, which are different from half-facial or full facial allografts that have been clinically tested (Figure 2). The corresponding nerves mainly involve the buccal and zygomatic branches of the facial nerve, the eye branch of the trigeminal nerve, and the GAN (Ulusal et al., 2005; Mathes et al., 2014; Gao et al., 2017). These studies confirmed the feasibility of using complex functional subunits for transplantation and provided more allograft options for complex craniofacial injuries.

In addition to difficulties in donor acquisition, craniofacial nerve allografts are associated with adverse consequences of long-term immunosuppression, especially infectious complications. Infection in craniofacial allografts follows a process similar to that of solid organ transplantation, but there are differences. The incidence of infectious complications was lower than that of solid organ transplantation (Roche et al., 2015a). Meanwhile, the graft, as a functional unit, has a unique source of bacterial flora. Craniofacial functional transplant units often include organs such as the oral cavity, nasal cavity, sinus, and upper respiratory mucosa, which may be sources of several pathogenic microorganisms. For instance, the oral mucosa can easily be colonized by *streptococcus*, *candida*, and several anaerobic bacteria. There is an increased risk of opportunistic infection if the graft contains sinuses or nasal mucosa that may contain fungal spores (Husain et al., 2003; Singh et al., 2005; Cuellar-Rodriguez et al., 2009). A mass of donor-derived bare skin may carry groups of pathogenic bacteria such as *staphylococcus*, gram-negative bacteria, and anaerobic bacteria (Gordon et al., 2011). This increases the requirements for the prevention of infections after transplantation. Therefore, it is necessary to develop a comprehensive program for infection prevention according to the type of functional unit used before transplantation. In conclusion, repairing complex craniofacial defects with an allogeneic composite functional unit has benefits and risks in craniofacial nerve transplantation. It can provide an ideal function and aesthetic effect that autologous transplantation cannot; however, it is also associated with facial immune rejection and the risk of infection caused by immunosuppression and special bacterial flora.

### Decellularized tissues

Decellularized tissues are obtained using physical, chemical, or enzymatic hydrolysis to destroy the bonds between cells and the extracellular matrix (ECM), remove the cellular components in the natural tissue, and retain the ECM, including key structural and functional proteins. This is associated with low immune rejection and provides a favorable microenvironment for cell growth. Therefore, decellularized tissue is considered a new graft for promoting nerve repair and regeneration. In essence, it can still be considered a type of allograft if the decellularized tissue is from the same species. Current studies confirm that decellularized tissue can remove components that inhibit the growth of nerve axons and retain growth-promoting proteins, which play a crucial role in promoting nerve repair (Buckenmeyer et al., 2020; Sun et al., 2020).

Although mature decellularization techniques for the sciatic nerve, spinal cord, and other tissues have been used to repair various nerve injuries, few attempts have been made to use decellularized tissues for craniofacial nerve repairs. Current decellularized products for clinical use are made from the purified ECM of human peripheral nerves after decellularization. It retains bundle structure and contains molecules crucial to the growth of axons, such as laminin (Hall, 1986). These decellularized tissues have been applied successfully to peripheral nerve regeneration with satisfactory effects as a mature substitute for nerve transplantation (Szynkaruk et al., 2013). There have been increased attempts to use decellularized tissues, with the main focus on the repair of trigeminal nerve injury in the field of craniofacial nerve transplantation (Zuniga, 2015; Salomon et al., 2016; Yampolsky et al., 2017). Yampolsky et al. supported the hypothesis that decellularized graft can be used to effectively repair short-distance (<2 cm) trigeminal nerve defects based on the outcomes of trigeminal nerve reconstruction (Yampolsky et al., 2017). Salomon et al. used decellularized nerve grafts as ECM scaffolds to repair the long-span (>30 mm) defects of the inferior alveolar nerve (IAN) and also observed good recovery after surgery (Salomon et al., 2016). In 2019, treated human decellularized allograft nerve was utilized to recover corneal perception in patients suffering from neurotrophic keratopathy by transplanting it to the supraorbital nerve, supratrochlear nerve, or IAN and transferring it to the affected eye. The study found that patients regained corneal perception and the integrity of corneal epithelial cells (Leyngold et al., 2019). However, some researchers have different perspectives, arguing that there is not sufficient evidence to support better outcomes with the use of decellularized tissue to repair long nerve defects than those of autografts or allografts (Moore et al., 2011; Giusti et al., 2012; Saheb-Al-Zamani et al., 2013; Poppler et al., 2016). The decellularized tissue loses SCs in during immunogenicity removal, which are critical cells for axon regeneration. SCs from the nerve remnant at the recipient site must fill the graft for nerve regeneration after implantation (Poppler et al., 2016). Therefore, the researchers do not recommend the use of current decellularized products to repair crucial nerves, long-diameter nerves, or short-diameter sensory nerves with gaps longer than 3 cm (Moore et al., 2009; Rbia and Shin, 2017). However, the potential advantages of decellularized tissues cannot be ignored in the field of craniofacial nerve transplantation and neural transplantation in its entirety. They eliminate the limitations of limited donor sources and damage to the donor site in autologous nerve transplantation. They also provide a good growth environment for nerve cells and reduce the risk of immune rejection and infection caused by other allotransplants. Recently, researchers have attempted to enhance the effectiveness of decellularized nerve tissues by combining them with cells or growth factors. For example, Schwann cells (SCs), adipose stem cells (ADSCs), and bone marrow mesenchymal stem cells (BMMSCs) can be combined in decellularized tissues. Compared with a single graft, decellularized tissue combined with cells or growth factors showed better outcomes in promoting nerve regeneration (Nadim et al., 1990; De Ugarte et al., 2003; Chen et al., 2007).

## Xenografts

There are no clinical applications of animal-derived xenografts for nerve repair. In skin transplantations, no skin substitutes can replace all the functions of intact human skin (Khan et al., 2020). However, xenotransplantation should still be considered given the shortage of donors and to reduce the injury to the donor site in autologous or allogeneic transplantation. For large soft tissue defects, autologous or allogeneic transplantation cannot meet the demand, and the need for xenotransplantation is more urgent. Therefore, research progress in xenotransplantation can promote xenotransplantation development in soft tissue injury nerve repair. At present, pigs are the main animal source of xenotransplantation tissue, with heart and kidney transplantation being the main operations (Hawthorne et al., 2021; Meier et al., 2021; Niu et al., 2021; Ryczek et al., 2021). The main disadvantage to xenotransplantation is antibody-mediated rejection (AMR). Based on previous research, the prevention and treatment of AMR include the following; preformed xenoantibodies are removed before transplantation, effectively avoiding hyperacute rejection (HAR) (Reichart and Längin, 2020). HAR is caused by the binding of preformed antibodies to porcine alpha 1,3- galactosyl- transferase (GAL). Similarly, complete knockout of the GAL gene in pig organs also prevents HAR. GAL gene-knockout pigs have been widely used in preclinical studies of xenotransplantation (Dai et al., 2002; Lai et al., 2002). With the development of gene editing technology, researchers have developed transgenic animals that express human complement regulators, such as CD40, to exert the immunomodulatory role of complement and thereby suppress immune rejection (Schmoeckel et al., 1996). Although systematic immunosuppressive therapy has achieved reliable efficacy, its serious side effects should not be ignored. Recent studies have suggested that immunological tolerance, based on regulatory T cells (Tregs), can significantly reduce the use of immunosuppressive agents (Romano et al., 2019). In conclusion, AMR and infection are still the primary problems that need to be faced and solved in future xenotransplantation for nerve repair of soft tissue injuries.

## Nerve conduits

Autologous and allogeneic nerve transplantations have achieved great success as common methods of nerve repair. However, surgical complications include nerve defects at the donor site, limited donor sources, and easy induction of infection. Therefore, fabricating various artificial nerve conduits based on tissue engineering as a substitute for autografts is currently an area of focus in nerve regeneration research (Long et al., 2021). The common nerve conduits in recent studies consist of autogenous conduits and artificial nerve conduits. Autogenous conduits include the blood vessels, muscles, tendons, and other autologous tissues. Previous studies have shown that veins and arteries can provide support for the damaged nerves. As tissues promote nerve repair, their effects are similar to those of the autografts (Chiu and Strauch, 1990; Tang et al., 1993). There are also differences between arteries and veins as nerve conduits. Veins are useful because of their abundance and lower incidence of donor-site complications. However, separate venous conduits are at risk of collapse and impeding nerve regeneration (Wolford and Stevao, 2003). Therefore, researchers have modified the veins by filling them with nerve or muscle tissue, which prevents the wall from collapsing and provides ECM and growth factors (Edgar et al., 1984; Lundborg et al., 1994). However, Tang JB et al. suggested that venous conduits were unsuitable for primary repair of nerves with nerve gaps greater than 5.0 cm or with complex nerve injuries based on their findings (Tang et al., 1995). Skeletal muscle contains multiple types of collagens (e.g., type IV collagen and laminin), which could guide the regeneration of the axon (Edgar et al., 1984; Lein et al., 1992; Lundborg et al., 1994; Meek et al., 2004). Therefore, skeletal muscle could be a good candidate for a nerve conduit, and it could assist in nerve regeneration as well as autologous nerve transplant (Pereira et al., 1991a; Pereira et al., 1991b; Meek et al., 2004). Tendons can also be used as autologous nerve conduits because they can provide laminin, fibronectin, and components that can promote the growth of axons (Guizzardi et al., 1987; Banes et al., 1988). Similar to muscle tissue, tendons can, theoretically provide a good spatial structure for nerve growth. However, previous studies have revealed that the ability of tendons to promote nerve regeneration has not met expectations (Brandt et al., 1999). Therefore, tendons are not preferred candidate material for autologous conduits in the field of nerve transplantation when compared to blood vessels and muscle.

In addition to autologous tissue, synthetic materials are the focus of research on nerve conduits. Multiple synthetic materials, such as polyhydroxy acid, collagen, polylactic acid glycolic acid, polycaprolactone, and silk, have been explored as substitutes for interposition transplants (Bini et al., 2004; Rinker and Liau, 2011; Taras et al., 2011; Huang et al., 2012; Reid et al., 2013; Sarker et al., 2018). However, although synthetic nerve conduits can provide some mechanical support, they lack the active ingredients that could promote axon growth. Recent studies have considered a combination of nerve conduits with special cells and growth factors that can promote axon growth or myelin formation. Olfactory ensheathing cells (OEC) are glial cells that secrete many neurotrophic factors to promote axonal growth. Based on this characteristic, Lee et al. implanted OECs into a nerve conduit and transplanted them into a rat sciatic nerve. The outcome revealed that there was no obvious difference in nerve conduction velocity, muscle wet weight, and nerve density between OEC-containing nerve conduits and autologous nerve grafts (Lee J. Y. et al., 2021). Other components commonly combined with nerve conduits include SCs, olfactory stem cells, ADSC, BMMSCs, IGF-1, and transforming growth factor-β (TGF-β) (Huard et al., 1998; Evans, 2001; Murrell et al., 2005; Wang et al., 2016a; Matsumine et al., 2016; Watanabe et al., 2017). Among them, induced pluripotent stem cells (iPSC) are a promising source of cells for nerve repair. They can be induced from adult somatic cells and, therefore, avoid ethical concerns (Takahashi and Yamanaka, 2006; Takahashi et al., 2007; Huang et al., 2020). Under suitable conditions, iPSCs can differentiate into SCs and promote axonal regeneration and myelination (Pan et al., 2022). They can also differentiate into neural crest-like cells that produce and secrete neurotrophic factors such as nerve growth factor (NGF) or VEGF, which could improve microcirculation and act directly on axons and endogenous SCs (Mittal and Schrenk-Siemens, 2020). However, iPSCs have an increased risk of uncontrolled cell division and tumorigenesis. Exosomes have functions similar to those of maternal cells but without immunogenicity. Therefore, in nerve repair, researchers use iPSC-derived exosomes to replace iPSCs, which not only retain the ability to promote nerve repair but also avoid the risk of carcinogenesis and other biosafety issues (Yu B. et al., 2014; Sun et al., 2016; Pan et al., 2022).

**FIGURE 3:**
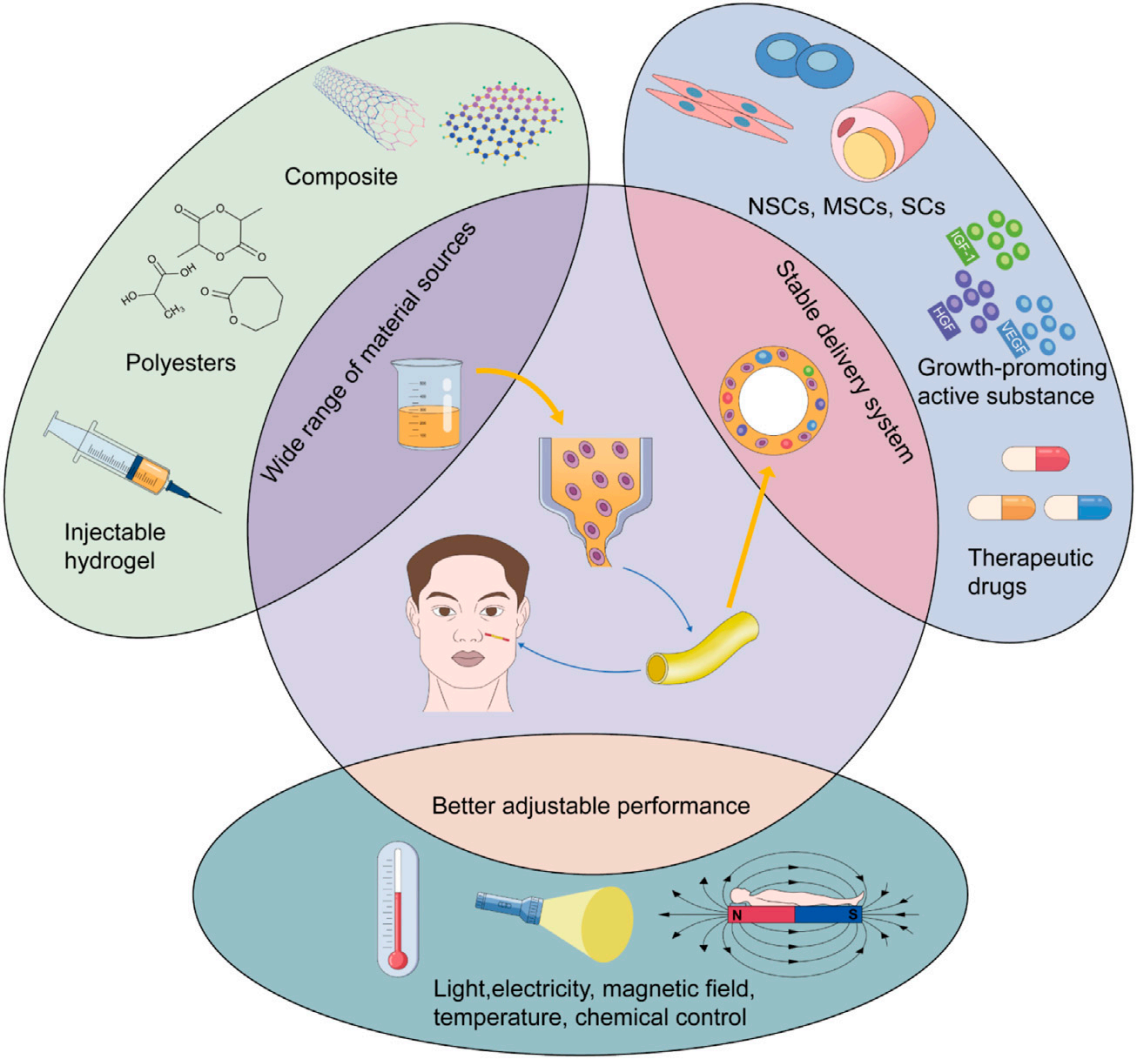
Advantages of 3D printing in craniofacial nerve transplantation.

Although synthetic conduits have shown unique advantages in peripheral nerve repair, they have not been widely applied in clinical practice. The conduits are still in the animal model stage, and in the field of craniofacial nerve transplantation research, the focus is on nerve injury repair in rats. For example, Long et al. cross-linked collagen tubes with heparin to construct a neural guidance pipeline for transporting nerve growth factor (NGF) and implanted it into facial nerve defects in rats. The short half-life of NGF was improved by the combination of NGF and collagen and was enhanced by heparin. Neural-guided conduits significantly promoted axonal growth and proliferation of SCs 3 months after surgery. The neurological recovery results were consistent with those of the autografts (Long et al., 2021). The characteristics of ADSCs are similar to the mesenchymal lineage and could differentiate into Schwann - like cells. ADSCs are easy to isolate and amplify. Based on these properties, a silicone tube containing ADSCs was inserted into the collagen gel and used to repair the gap in the rat nerve. Postoperative examination showed that the nerve repair ability of the synthetic material was nearly identical to that of the autologous nerve transplants (Watanabe et al., 2017). Therefore, in the field of craniofacial nerve repair, the effect of a nerve conduit is positive in animal models, especially in facial nerve injury repair. However, it is still necessary to research its application in other nerves, such as the trigeminal and glossopharyngeal nerves and other nerves that have important sensory or motor functions and are vulnerable to damage. However, more cases are needed to verify its clinical feasibility. Attention should be paid to nerve conduits, which have great potential as substitutes for autografts. Moreover, the rapid improvement in 3D printing has promoted the technological progress of nerve conduits. The 3D printing technology allows for the rapid fabrication of complex peripheral nerve conduits and enables multiple types of materials to be involved in the synthesis of the conduits, such as hydrogels, thermoplastics, thermosetting polyesters, and bioinks. This could provide more physiologically suitable grafts for nerve repair and a wider delivery system for precursor cells and biologically active substances (Ashraf et al., 2019; Yu et al., 2020; Bahremandi Tolou et al., 2021). Researchers have even introduced the concept of 4D printing into neural repair. They have proposed inducing dynamic reactions of the 3D-printed scaffold using temperature, pH, light, electricity, and other stimuli. This can realize intelligent control of the internal environment of the recipient site and regulate the differentiation and migration of nerve cells. Combined with these stimulation factors, the performance of synthetic conduits can be improved to regulate nerve repair better under physiological conditions (Zhu et al., 2017; Fantino et al., 2018; Uz et al., 2019) (Figure 3). In conclusion, it is worth exploring the important role of the conduit as an alternative to the autograft in craniofacial nerve transplantation and even in the field of peripheral nerve repair as a whole. Further research on the laboratory to clinical transformation of this technique is worthwhile.

## Novel materials

The nerve conduit is aimed at replacing autografts. However, the repair of long nerve defects remains limited. Bioengineering and nanomedicine focus on the construction of bioactive materials and the surface modification of neural conduits or 3D biological scaffolds to promote the attachment and survival of cells and molecules. This could effectively promote the long-distance regeneration of axons (Sun et al., 2010; Pabari et al., 2014; Faroni et al., 2015; Mobasseri et al., 2015). Hydrogels and nanomaterials are two novel materials with the most potential for nerve repair. They are possible replacement materials for craniofacial nerve transplantation and the combined repair of craniofacial soft tissues because of their superior biocompatibility and stimuli-responsiveness.

### Hydrogels

Hydrogels can be classified in several ways. Here, they are divided into natural and synthetic hydrogels, according to their source. Hydrogels are perfect carriers of active substances and are biodegradable. They can, therefore, provide structural and nutritional support for the growth of neural tissues in the early stage of repair but also provide suitable space for the growth of newly formed tissue through gradual degradation. Moreover, it has satisfactory biocompatibility compared with other materials. It can provide a growth environment similar to the extracellular matrix for nerve repair and guide the orderly regeneration of axons in specific directions, helping to rebuild damaged neural networks (Araújo et al., 2017; Celikkin et al., 2017; Fan et al., 2017) (Table 2). Natural hydrogels include hyaluronic acid (HA), fibrin, chitosan, and silk fibroin. HA can hydrate the ECM and regulate the dynamic balance of tissues as a polysaccharide component of the extracellular matrix (ECM). It also helps the matrix maintain the gel state and promotes the structural stability of the ECM by interacting with other proteins or polysaccharides (Abatangelo et al., 2020). Cross-linked HA hydrogels have been shown to promote significant axonal growth and to achieve sustained electrical activity and strong synaptic connections *in vivo* (Horn et al., 2007; Broguiere et al., 2016). Meanwhile, HA hydrogel scaffolds have also shown advantages in neural regeneration, including improving the survival rate of NSCs and SCs and promoting the differentiation of neural progenitor cells into neurons and glial cells. However, this property can be affected by various chemical modifications (Pan et al., 2009; Seidlits et al., 2010; Liang et al., 2013; Wang et al., 2013). Porous cross-linked HA hydrogels provide the possibility of accelerating nerve regeneration by promoting cell infiltration, assisting in angiogenesis, and inhibiting scar formation during repair (Hou et al., 2005). In line with HA hydrogels, other types of natural hydrogels, such as fibrin, collagen, chitosan, and keratin hydrogels, are also highly effective in promoting the differentiation of NSCs and axonal regeneration (Sierpinski et al., 2008; Mooney et al., 2010; Pace et al., 2014; Yao et al., 2016; Salehi et al., 2018; Yoo et al., 2020). They do, however, exhibit different properties due to their diverse composition. Degradation products such as chitosan hydrogels can protect nerve cells from toxic and oxidative damage and stimulate the proliferation of SCs (Zhou et al., 2008; Huang et al., 2015; Wang et al., 2016b). Sericin has significant neurotrophic effects as a kind of natural protein. Glycine and serine are degradation products of sericin and are important neurotransmitters that play indispensable roles in information transmission (Hernandes and Troncone, 2009).

**TABLE 2:** The common hydrogel materials in nerve repair.

Materials	Additives	Main outcomes	Target cells/tissue	Model	Ref.
Hyaluronic acid cross-linked by galactose oxidase and horseradish peroxidase	BMMSC, NGF	Providing nutrition supply for cell survival and proliferation and suppressing neuroinflammation and apoptosis	Neural cells in the hippocampus	Traumatic brain injury in mice	Wang et al. (2022)
Hyaluronic acid cross-linked by transglutaminase	-	Showing fast neurite outgrowth, strong synaptic connectivity, and long-lasting coordinated electrical activity	Embryonic neurons from rats	-	Broguiere et al. (2016)
Chitosan hydrogel	BDNF, VEGF	Promoting the proliferation and secretion of neurotrophic factors by Schwann cells and vascular penetration	Schwann cells from rats	Sciatic nerve defects in rats	Rao et al. (2020)
Double cross-linked chitosan hydrogel	-	Promoting Schwann cell proliferation and sciatic nerve regeneration	Schwann cells from rats	Sciatic nerve defects in rats	Deng et al. (2022)
P-conjugated chitosan hydrochloride hydrogel	-	Accelerating full-thickness wound healing by enhancing synchronized vascularization, extracellular matrix deposition, and nerve regeneration	HUVECs, fibroblasts, and Neuro-2A cells	Full-thickness skin wounds of rats	Li et al. (2021a)
Collagen hydrogels	MSC	Promoting neuronal differentiation and suppressing inflammatory reaction	Neural stem cells from the embryonic brain of rats	-	He et al. (2021)
XT-type DNA hydrogels	VEGF, NGF	Promoting proliferation, migration and myelination of Schwann cells	Schwann cells from rats	Sciatic nerve defects in rats	Liu et al. (2021)
Extracellular matrix hydrogel	-	Promoting increased macrophage invasion, higher percentages of M2 macrophages and enhanced Schwann cell migration	-	Sciatic nerve defects in rats	Prest et al. (2018)

In addition to natural materials, synthetic hydrogels have been widely used for nerve regeneration. Synthetic materials have strong plasticity and superior physical properties compared to natural materials. Polyethylene glycol hydrogel is an adhesive gelatinous material that acts as a fibrin sealant to enhance nerve repair. It can effectively reduce scar formation and contribute to axonal regeneration and myelination (Isaacs et al., 2009; Estrada et al., 2014). Hejcl et al. found that the positively charged 2-hydroxyethyl methacrylate (HEMA) hydrogel was structurally conducive to massive implantation of connective tissue. They observed that the porous structure of the 2-MEMA hydrogel was filled with blood vessels, nerve filaments, and Schwann cells, making it a good scaffold for axon growth (Hejcl et al., 2008). Although synthetic hydrogels have advantages in their physicochemical properties, they are inferior to natural materials in biocompatibility. Therefore, it is necessary to improve biocompatibility through the molecular modification of synthetic hydrogels or by fusing them with other materials. For example, researchers modified poly (2-hydroxyethyl methacrylate) using laminin-derived peptides to improve cell adhesion and promote the differentiation of NSCs (Kubinová et al., 2010). In conclusion, hydrogels have obtained positive results in the field of neural repair because of their good biocompatibility and plasticity. Adjusting the physicochemical properties of hydrogels to improve their nerve repair ability or using them as cellular or molecular carriers to promote nerve regeneration is still an important research direction. Research on the comprehensive ability of hydrogels to repair nerves, blood vessels, muscles, and skin is in great demand for craniofacial nerve transplantation and craniofacial multi-tissue combined repair.

### Nanomaterials

Nanomaterials have inherent advantages for tissue repair. First, nanomaterials can be used as carriers of a wide range of drugs, bioactive substances, or genes because of their high surface-area-to-volume ratio (Kim and Hyeon, 2014). Secondly, the stimuli responsiveness is the intrinsic characteristic of nanometer materials. These materials can respond to changes in stimuli, including pH, temperature, light, and magnetic fields. In addition, the strength, surface morphology, and degradation behavior of nanomaterials can be regulated by different molecular or synthetic conditions to meet the requirements of tissue repair. In the field of nerve repair, nanomaterials do not only serve as fillers for nerve conduits to provide structural and nutritional support (Li et al., 2014; Meyer et al., 2016) but their inherent properties, such as conductivity, can also be taken advantage of to improve the physical and chemical properties of nerve conduits (Oprych et al., 2016). Meantime, nanoparticles can deliver molecules to cells in a directional manner (Chen et al., 2017). This section reviews the common nanomaterials and their properties in nerve repair to promote their application in craniofacial nerve transplantation and craniofacial repair. Nanoscale hydrogels were not repeated.

Carbon nanotubes (CNTs) are chemically stable and inert. They have electrical properties consistent with those of neural tissue. They also facilitate cell adhesion, differentiation, and growth. These characteristics support their application in neural repair (Hopley et al., 2014; Alshehri et al., 2016). CNTs have shown the general characteristics of repair materials, such as the ability to promote nerve cell differentiation and axon growth (Yu W. et al., 2014; Roberts et al., 2014; Ahn et al., 2015). They also show advantages in promoting nerve regeneration. For example, using carbon nanotubes as coatings for polydimethylsiloxane (PDMS) scaffolds enhances cell adhesion and viability while doubling the survival and proliferation rates of primary neurons and SCs (Kang et al., 2015). CNTs are superior conductive materials. Their conductive properties and unique nanoscale textures may affect the growth of cells and neurites. Researchers have found that long neurites tend to line up in the direction of the nanotubes on horizontal CNTs, which has important implications for using physical and electrical factors to direct axonal growth (Roberts et al., 2014). Tu Q et al. observed the effect of surface charge on neurites and its branches. The results showed that positively charged graphene oxide (GO) was more conducive to the growth and branching of neurites than neutral ions, zwitterions, or negatively charged GO (Tu et al., 2014). The application of metal nanoparticles, such as iron, gold, and silver, has attracted more attention in nerve repair. Superparamagnetism and photoreactivity are the two characteristics that distinguish metal nanoparticles from other materials. Superparamagnetic nanoparticles (SMNPs) can guide cells to specific locations by using an external magnetic field. This superparamagnetism can contribute to the directional growth of neurites during nerve repair (Riggio et al., 2014; Marcus et al., 2016). The distribution of nerve cells and the growth direction of primary neurons can be successfully regulated by controlling the external magnetic field gradient using magnetic iron nanoparticles as a medium. Therefore, as a remote control platform, magnetic nanoparticles (MNPs) are expected to become a new therapeutic agent for nerve injury (Marcus et al., 2016). Gold nanoparticles are widely used in biomedical research for their unique optical properties, as their absorption spectrum can vary in the range between visible and near-infrared. Irradiation of gold nanorods in NG108-15 neural cells with a 780 nm laser stimulated cell growth, especially neurite growth (Paviolo et al., 2013). Similarly, stimulation of gold nanorod-treated neural cells with 780 nm near-infrared light-induced intracellular calcium transients. This stimulatory effect may be related to the mechanisms of cell differentiation and axon growth under optical induction (Paviolo et al., 2014). Recently, the combination of nanomaterials and hydrogels has shown its advantages. Hybrid materials possess the biological affinity of hydrogels and the superior photoelectric, mechanical, thermal, and other physical properties of nanomaterials (He et al., 2020; Huang et al., 2021; Zheng et al., 2022). For example, combining CNTs with self-assembled peptides had better electrical conductivity and injectable properties, providing a practical method for promoting nerve repair (He et al., 2020). The fusion of pegylated CNTs with silk fibroin provided a flexible scaffold for photoacoustic nerve stimulation (Zheng et al., 2022). Table 3 summarizes the recent studies on nanomaterial binding hydrogels in peripheral nerve regeneration.

**TABLE 3:** The common researches of nanomaterial binding hydrogel in peripheral nerve regeneration.

Nanomaterial	Hydrogel material	Main outcomes	Cell model	Animal model	Ref.
Carbon nanotubes	Functional self-assembling peptide	Promoting axon growth and myelination	Dorsal root ganglia neurons from rats	-	He et al. (2020)
Polyethylene glycol-functionalized carbon nanotubes	Silk fibroin	Demonstrating nongenetic photoacoustic neural stimulation functions and promoting neurite outgrowth	Cortical neuron and dorsal root ganglia from rats	Skin injury model in mice	Zheng et al. (2022)
Poly (3,4-ethylenedioxythiophene) nanoparticles	Chitin	Enhancing angiogenesis and the proliferation of Schwann cells	Schwann cells from rats	Sciatic nerve defects in rats	Huang et al. (2021)
Poly (L-lactic acid)	Decellularized matrix from porcine sciatic nerves	Directing and promoting axonal extension, nerve fiber myelination, and functional recovery	Dorsal root ganglia from rats	Sciatic nerve defects in rats	Zheng et al. (2021)
PHBV-magnesium oleate-N-acetyl-cysteine	Gellan/xanthan	Simulating a neuronal microenvironment conducive of axonal repair, particularly in the early stages of nerve regeneration	Rat pheochromocytoma cells	Sciatic nerve defects in rats	Ramburrun et al. (2021)
Polycaprolactone	Collagen/hyaluronic acid	Enhancing the proliferation of Schwann cells and axonal growth	Schwann cells and dorsal root ganglia from rats	-	Entekhabi et al. (2021)
Polycaprolactone	Sodium Alginate cross-linked with N,N′-disuccinimidyl carbonate	Increasing the number of myelinated axons and Schwann cell migration	Rat pheochromocytoma cells	Sciatic nerve defects in rats	Askarzadeh et al. (2020)

Recently, the advantages of nanoencapsulation have become increasingly apparent. Its application in neural repair is a topic of great interest. Nanoencapsulation uses an ultrathin external structure (<100 nm) to encapsulate viable cells or factors (Ariga and Fakhrullin, 2021). External materials come from a wide range of sources, such as polymers, hydrogels, and minerals (Lee H. et al., 2021). Its main advantage is its better physicochemical protection, which allows cells and factors to survive and function under harsh conditions such as ultra-low temperatures and toxic microenvironments (Park et al., 2014; Youn et al., 2017; Zhu et al., 2019; Wang et al., 2021). However, recent studies have extended the benefits of nanoencapsulation. Based on a metal-organic framework, Lee et al. combined Fe3+ and benzene-1,3, 5-tricarboxylic acid to wrap living cells. Thus, the catalytic efficiency of the enzymatic reactions was improved. Nanoshells have exogenous biochemical functions, such as converting toxic chemicals into nutrients (Lee et al., 2022). Polyphenols have strong anti-inflammatory properties, but their low bioavailability limits their application. The nano-embedding of polyphenols with different carriers can improve their bioavailability and reduce their degradability (Jayusman et al., 2022). In addition, as a natural barrier in the human body, the blood-brain barrier prevents harmful substances from entering the brain and also hinders the entry of most drugs. Lipid-based nanoparticles are good carriers for neuroprotective drugs and factors that cross the blood-brain barrier (Fernandes et al., 2021). In conclusion, we believe that nanoencapsulation can show its unique advantages in future neural repair research. Furthermore, the use of nanoencapsulation to create a system composed of physically separated but interacting cell hybrids is also a future research direction.

Although a variety of nanomaterials have shown potential in promoting nerve repair, their toxicity to cells and tissues cannot be ignored. These side effects arise mainly from two aspects: Reactive oxygen species (ROS) may be generated during the interactions between nanoparticles and cells. This leads to oxidative stress, which in turn threatens the growth of cells and tissues (Soto et al., 2007; Balasubramanyam et al., 2009; Radziun et al., 2011). However, the toxicity does not originate from the nanoparticles themselves but from the coating applied in the process of surface modification and functionalization (Rivet et al., 2012). In addition, there are specific side effects associated with different material types. CNTs have been shown to have effects on the reproductive system, affecting embryonic development and delaying pregnancy. These side effects have been proven to be concentration-dependent (Philbrook et al., 2011; Xu et al., 2015). In addition to causing oxidative stress, silver nanoparticles are prone to deposition in organs such as the kidney, liver, spleen, and lungs. In animal testing, it has been found to penetrate the blood-brain barrier and enter nerve cells, causing neuronal degeneration (Tang et al., 2009). Therefore, the neurotoxicity of nanomaterials should be fully considered in the selection, surface modification, and functionalization of these conduits.

## Summary

Although there are many surgical methods and grafts for nerve repair in craniofacial soft tissue injuries, the ultimate purpose is to achieve functional and aesthetic normalization. Autografts are still the preferred choice for segmental nerve defects in clinical practice because their regeneration microenvironment is the most suitable for physiological conditions (Moore et al., 2015). Vascularization of the graft can promote an immediate postoperative blood supply and achieve better functional recovery compared to nerve grafts alone (Koshima et al., 2004; Li S. S. et al., 2021). At the same time, when it comes to motor nerve transfers, dual nerve transfers and combined transplantation of sensory and motor nerves are effective methods to reduce postoperative mass movement (Yoshioka, 2018; Kuta and Taylor, 2022). But the trauma and nerve defect complications at the donor site are inevitable. However, with the development of tissue engineering technology, artificial tissues or scaffolds may replace autografts. For complex craniofacial defects, using allogeneic composite functional units for transplantation is inevitable to achieve functional recovery (Zor et al., 2010; Roche et al., 2015a). The nerve tissue in this unit plays a major role in functional recovery, while the effect of the structural restoration of bone and other soft tissues is also indispensable. However, the limited source of donors and individual differences are inherent problems. Immunosuppression and infection have puzzled researchers for a long time. In particular, the complexity of cranial and facial tissues increases the risk of infection by specific bacterial flora from multiple sources (Husain et al., 2003; Singh et al., 2005; Cuellar-Rodriguez et al., 2009). Therefore, designing more reasonable allogeneic composite functional units by using animal models and controlling the infection during perioperative and early postoperative periods are urgent areas of study that need to be explored. Decellularized tissue is rarely rejected because of the removal of immunogenicity and has been widely used in peripheral nerves. However, decellularized tissue has not shown better results for functional recovery than autografts in clinical studies of the craniofacial nerves. Therefore, it should be applied with caution in large-caliber nerves and long defect gaps (Moore et al., 2009; Rbia and Shin, 2017). But its advantages of low immunogenicity and the absence of donor site complications should not be overlooked. Therefore, combining decellularized tissue with special additives (e.g., NSCs and growth factors) to produce new grafts that are more suitable for the physiological environment of nerve regeneration is an important part of current research. Traditional autologous nerve conduits (e.g., arteries, veins, muscles, and tendons) have shown good results in promoting nerve repair in clinical applications, but they do not show stronger repair ability than autologous nerve grafts and have defects leading to complications at the donor site (Chiu and Strauch, 1990; Tang et al., 1993; Tang et al., 1995; Brandt et al., 1999; Meek et al., 2004). Synthetic conduit materials have a wide range of sources and strong plasticity. Synthetic conduits have more physiologically appropriate structures and have become better delivery systems for a wide range of cells, growth factors, and drugs because of advances in tissue engineering, particularly 3D printing (Ashraf et al., 2019; Yu et al., 2020; Bahremandi Tolou et al., 2021). However, this technique has not been widely applied in nerve transplantation. Therefore, research to clinical transformation of synthetic conduits combined with 3D printing technology is undoubtedly the focus of current research. With the introduction of 4D printing, basic research should focus on achieving dynamic regulation of 3D scaffolds using stimulation factors such as temperature, pH, light, and electricity. This is conducive to the construction of new materials that can intelligently adjust the nerve repair microenvironment and promote nerve function normalization. The replacement of biological grafts with synthetic materials is new in the field of nerve transplantation. As representative novel materials, hydrogels and nanomaterials show excellent biocompatibility and stimuli-responsiveness (Kim and Hyeon, 2014; Araújo et al., 2017; Celikkin et al., 2017; Fan et al., 2017). Their ability to promote neural cell proliferation, inhibit scar formation, and accelerate the growth of neurites has been demonstrated. However, the toxicity and side effects (on the reproductive system and oxidative stress) of the material and modifying substances should be avoided. Future research should focus on improving and modifying nerve conduits using novel materials to promote the directional growth of axons and inhibit scar formation. This may solve the problem of repairing long nerve defects.

## Fundings

This work was supported by grants from the National Natural Science Foundation of China (82172740, 82111530202, 81874082), Shandong Provincial Natural Science Foundation (ZR2021LSW008, ZR2020QH234), Clinical Research Center of Shandong University (No. 2020SDUCRCB002), Department of Science & Technology of Shandong Province (ZR2019ZD33 and 2020CXGC010903), Special Foundation for Taishan Scholars (ts20110814, tspd20210322), Research Project of Jinan Microecological Biomedicine Shandong Laboratory (JNL-2022003A and JNL-2022042C), Innovation Project of Jinan Science and Technology Bureau (2021GXRC065), and Rongxiang Regenerative Medical Fund (2019SDRX-19).

## Abbreviations

ADSC, adipose stem cell; BDNF, brain-derived neurotrophic factor; BMMSC, bone marrow mesenchymal stem cell; CNT, carbon nanotube; ECM, extracellular matrix; EPO, erythropoietin; GAN, great auricular nerve; GO, graphene oxide; HA, hyaluronic acid; IAN, inferior alveolar nerve; IGF-1, insulin-like growth factor-1; MSC, mesenchymal stem cell; NGF, nerve growth factor; NSC, neural stem cell; OEC, olfactory ensheathing cell; ROS, reactive oxygen species; SC, Schwann cell; SCM, sternocleidomastoid muscle; TGF-β, transforming growth factor-β; VEGF, vascular endothelial growth factor.
